# Zerumbet Ginger Extract Attenuates Chemotherapy‐Induced Toxicity and Promotes Tumor Suppression in a Murine Breast Cancer Model

**DOI:** 10.1002/fsn3.71273

**Published:** 2025-11-25

**Authors:** Li‐Yu Su, Liang‐Chuan Lai, Chi‐Feng Cheng, Chia‐Ying Lien, Ming‐Chung Lee, Wu‐Chang Chuang, Chung‐Hsin Wu

**Affiliations:** ^1^ Department of Physiology, College of Medicine National Taiwan University Taipei City Taiwan; ^2^ Department of Oncology Taipei City United Hospital, Renai Branch Taipei City Taiwan; ^3^ National Taiwan University Taipei City Taiwan; ^4^ Brion Research Institute of Taiwan New Taipei City Taiwan; ^5^ School of Life Science National Taiwan Normal University Taipei City Taiwan

**Keywords:** antioxidant activity, breast cancer, chemotherapy, doxorubicin, TRPM2 channel, zerumbet ginger extract

## Abstract

Zerumbet ginger extract (ZGE) shows strong anticancer potential and enhances doxorubicin (DOX) efficacy against breast cancer while reducing its systemic toxicity. GC–MS identified zerumbone as a key bioactive. ZGE inhibited 4 T1 cell growth and tumor progression in mice and improved antioxidant activity. Combined ZGE + DOX treatment boosted antitumor effects and alleviated DOX‐induced cardiotoxicity, hepatotoxicity, and nephrotoxicity, as reflected in biochemical markers. Mechanistically, ZGE suppressed TRPM2 and calcium signaling, promoting ROS‐mediated apoptosis. These findings support ZGE as a promising adjuvant in breast cancer therapy.

## Graphical abstract

Zerumbet ginger extract (ZGE) enhances doxorubicin's anticancer effects while reducing its toxicity to the heart, liver, and kidneys in a murine breast cancer model. ZGE suppresses TRPM2‐mediated calcium signaling and promotes ROS‐induced apoptosis, contributing to tumor inhibition. These findings support ZGE as a safe and effective adjuvant for improving chemotherapy outcomes.

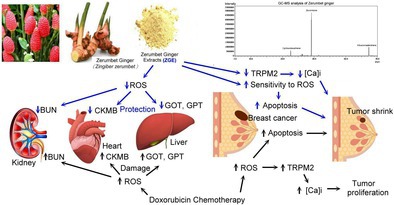

## 1 Introduction

Breast cancer remains a leading cause of cancer‐related death among women worldwide (Siegel et al. 2022). Triple‐negative breast cancer (TNBC), lacking ER (estrogen receptor), PR (progesterone receptor), and HER2 (human epidermal growth factor receptor 2) expression, presents a major therapeutic challenge due to its aggressive nature and poor prognosis. Even in estrogen receptor‐positive cases, resistance to hormonal therapy is not uncommon, underscoring the limitations of current treatment options.

Chemotherapy remains a cornerstone in breast cancer treatment. Doxorubicin (DOX) is a widely used chemotherapeutic agent for breast cancer, known for its ability to induce reactive oxygen species (ROS) accumulation, DNA damage, and apoptosis. DOX‐induced oxidative stress activates NF‐κB signaling, which plays a key role in myocardial differentiation and inflammation, contributing to cardiovascular toxicity (Xu et al. 2020). However, its clinical utility is constrained by dose‐dependent toxicity, particularly cardiotoxicity, including cardiomyopathy, arrhythmia, and heart failure risk (Adel 2017; Bisht et al. 2025). Thus, mitigating DOX toxicity without compromising its anticancer efficacy is an urgent clinical priority. Natural compounds with antioxidant and anti‐inflammatory properties have gained attention as potential adjuvants.

*Zingiber zerumbet* , a medicinal ginger species, yields extracts rich in zerumbone with anticancer, anti‐inflammatory, and cardioprotective effects (Dehghan et al. 2021; Radaei et al. 2020). This study innovatively investigates whether ZGE can enhance DOX's anticancer effects while reducing its cardiotoxicity and systemic side effects. Recent studies have demonstrated elevated expression of transient receptor potential melastatin 2 (TRPM2) channels in breast tumor tissues (Ali et al. 2023). We also evaluate ZGE's ability to inhibit TRPM2‐mediated calcium signaling, thereby disrupting survival and anti‐apoptotic pathways in breast cancer cells. Through integrated cellular and animal models, we aim to demonstrate ZGE's dual potential: potentiating chemotherapy and protecting healthy tissues, offering a novel and translationally relevant strategy for breast cancer therapy.

## 2 Materials and Methods

### 2.1 Preparation of ZGE

Zerumbet ginger extract (ZGE) was prepared following the method described by Ghasemzadeh et al. (2017). Zerumbet ginger ( *Zingiber zerumbet* ) was authenticated by the botanist of Brion Research Institute of Taiwan. The test samples conformed to the macroscopic and microscopic identification criteria for Red Ball Ginger as specified in the Thai Herbal Pharmacopeia. The reference standard material is stored at the Brion Research Institute of Taiwan. Fresh rhizomes of *Zingiber zerumbet* were obtained from Sun Ten Pharmaceutical Company (New Taipei City, Taiwan), thoroughly washed, and dried in a hot‐air oven at 55°C until constant weight. The dried rhizomes were ground into fine powder and sieved through a 60‐mesh filter. Approximately 100 g of powder was extracted with 1 L of 70% ethanol under ultrasonic agitation at room temperature for 3 h. The extract was filtered, concentrated using a rotary evaporator at 45°C, freeze‐dried, and stored at −20°C until use.

### 2.2 GC–MS and DPPH Assays of ZGE

**FIGURE 1:**
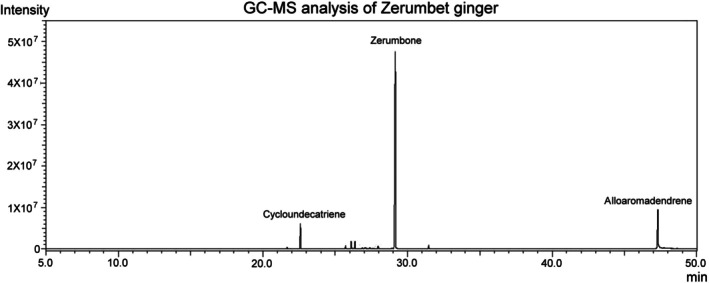
GC–MS analysis of ZGE revealed key bioactive compounds—including Zerumbone, Cycloundecatriene, and Alloaromadendrene—detected between 5 and 50 min. These active compounds are volatile constituents specific to ZGE. These peaks confirm the extract's chemical diversity and therapeutic potential.

Gas chromatography–mass spectrometry (GC–MS) analysis of ZGE was performed by HERBIOTEK Co. Ltd. (New Taipei City, Taiwan) to identify and quantify its major phytochemical constituents. The ZGE powder (1 g) was extracted with 10 mL of ethyl acetate through ultrasonic oscillation at 25°C for 20 min. The solution was concentrated under reduced pressure, and the concentrated material was resolved in 1 mL of ethyl acetate. The Shimadzu GC/MS analysis system consisted of a Shimadzu GC‐2010 gas chromatograph, Shimadzu GCMS‐QP2010 mass spectrometer and Shimadzu AOC‐20i + s autosampler. An Rtx‐5MS (30 m × 0.25 mm ID × 0.25 μm df, Restek) was used as the stationary phase and helium was the carrier gas. The total program time was 40 min, starting at 40°C for 2 min and increasing temperature at 5°C/min. The injection volume was 1 μL and the mass range was 45–450 m/z. Results were identified by NIST17/FFNSC1.2/WILEYB Mass Spectral Library with a Similarity index (SI) over 90%. The relative concentration of each component (peak) was calculated by dividing the peak area by the total area multiplied by 100%. As shown in Figure 1, the major compound in ZGE is zerumbone, which appears at approximately 30 min and exhibits the highest peak intensity. Other identified compounds include cycloundecatriene (around 20 min) and alloaromadendrene (around 45 min), indicating the chemical diversity of the extract. GC–MS standards are typically pure compounds that match the bioactive markers targeted for identification and quantification. Compared to liquid‐phase separation using HPLC, ZGE components are more effectively resolved in the gas phase using GC–MS, especially for volatile and semi‐volatile compounds. The chemical standard of zerumbone was purchased from Sigma‐Aldrich (USA) and used to confirm retention time and quantify concentration, ensuring analytical accuracy and reproducibility.

The antioxidant capacity of ZGE was assessed using the DPPH (α, α‐diphenyl‐β‐picrylhydrazyl) radical scavenging assay. On the basis of solubility and previous antioxidant screening protocols (Cheng et al. 2023), ZGE solutions at concentrations of 0.1, 0.5, 1, 3, 5, 10, and 20 mg/mL were individually mixed with 100 μL of 1.5 mM DPPH solution (Sigma‐Aldrich, D9132) in a 96‐well microplate. After gentle vortexing, samples were incubated in the dark at room temperature for 30 min. Absorbance was measured at 517 nm using a microplate spectrophotometer (μQuant, BioTek, USA). Ascorbic acid (Sigma‐Aldrich, A5960) served as a positive control. Blanks included ZGE only, DPPH only, and methanol. Scavenging activity (%) was calculated using the formula: Scavenging activity (%) = 100% × [(ZGE + DPPH absorbance) − (ZGE blank)]/[(DPPH absorbance) − (methanol blank)].

### 2.3 4 T1 Cells Preparation

The 4 T1 mouse breast cancer cell line, derived from a spontaneous tumor in BALB/c mice, was obtained from the Bioresource Collection and Research Center (Hsinchu, Taiwan) and used to model triple‐negative breast cancer. As we previously reported (Lu et al. 2021), cells were cultured in RPMI‐1640 medium (Gibco) supplemented with 10% fetal bovine serum and 1% penicillin–streptomycin, maintained at 37°C in a humidified 5% CO_2_ incubator. They exhibited adherent epithelial‐like morphology and a doubling time of 14–16 h. At 80%–90% confluence, cells were detached using 0.25% trypsin–EDTA for 2–5 min, neutralized with fresh medium, centrifuged, resuspended, and seeded at a 1:4 ratio. All experiments were performed using cells between passages 3 and 10 to ensure phenotypic stability and reproducibility.

### 2.4 Animal Preparation

**FIGURE 2:**
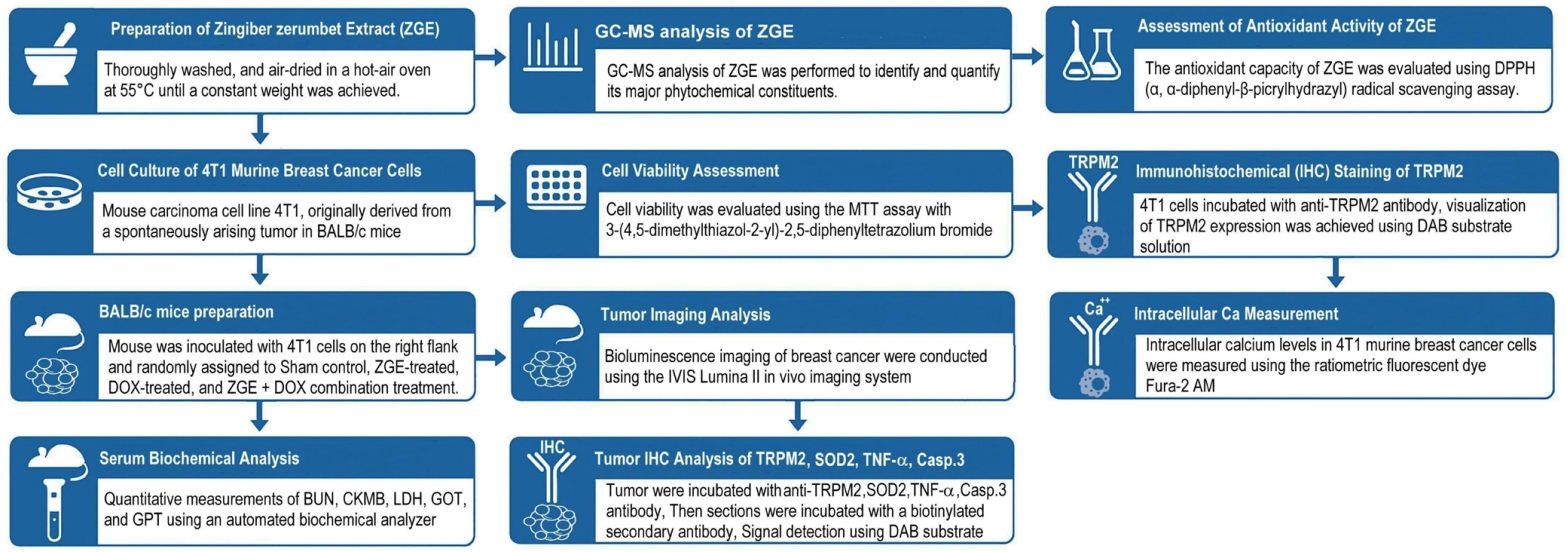
Experimental workflow evaluates ZGE's effects on breast cancer using cell assays, GC–MS profiling, and mouse models. It tracks tumor growth, organ toxicity, and molecular changes (TRPM2, Ca^2+^, apoptosis) to assess therapeutic potential and synergy with DOX.

In this study, 32 20‐week‐old female BALB/c mice were purchased from BioLASCO (Yilan, Taiwan) and housed at 22°C ± 2°C under a 12 h light/dark cycle with free access to chow and water. All procedures followed international animal care guidelines and were approved by the Institutional Animal Care and Use Committee of National Taiwan Normal University (Approval No. NTNU/Animal Use/No. 110024), adhering to the 3Rs principle (Replacement, Reduction, Refinement) to ensure animal welfare. The experimental workflow is shown in Figure 2. For in vivo experiments, 5 × 10^6^ 4 T1 breast cancer cells were injected into the right flank of BALB/c mice to establish a tumor model. According to Mohd Salleh et al. (2024), the acute toxicity study showed that ZGE is safe at the highest 5000 mg/kg body weight dose and treated with ZGE at doses of 125, 250, and 500 mg/kg in Sprague Dawley rats. In this study, groups included sham control, ZGE oral treatment (150 mg/kg, twice daily for 4 weeks), DOX intraperitoneal treatment (2.4 mg/kg, every 2 days for 4 weeks), and ZGE + DOX combination therapy. Tumor progression was monitored using the IVIS Lumina II bioluminescence imaging system. The regulatory effect of ZGE on TRPM2‐Ca^2+^ signaling in tumor tissue was examined. Serum markers (BUN, CKMB, LDH, GOT, GPT) were measured to assess liver, kidney, and heart function.

### 2.5 ROS, MTT and Intracellular Ca^2+^ Assays of 4 T1 Cells

To evaluate ZGE's antioxidant effect in 4 T1 breast cancer cells, intracellular ROS levels were measured. Cells were seeded in 96‐well plates (5 × 10^4^ cells/well) and incubated overnight at 37°C with 5% CO_2_. After 24 h treatment with ZGE, cells were incubated with 10 μM DCFH‐DA for 30 min in the dark. ROS oxidizes DCFH‐DA to fluorescent DCF, which was measured at 485/535 nm. Ascorbic acid (100 μM) was used as a positive control; untreated cells served as the baseline. ROS levels were expressed as relative fluorescence units (RFU) and normalized. ZGE reduced ROS accumulation in a dose‐dependent manner, indicating strong intracellular antioxidant activity.

Cell viability was assessed using the MTT assay with 3‐(4,5‐dimethylthiazol‐2‐yl)‐2,5‐diphenyltetrazolium bromide (MTT; Sigma‐Aldrich, M5655). 4 T1 murine breast cancer cells were seeded in 96‐well plates at 5 × 10^5^ cells/mL and incubated for 24 h at 37°C under 5% CO_2_. On the basis of solubility and previous antioxidant screening protocols (Cheng et al. 2023), cells were treated with ZGE (0.1, 1, 3, 5 mg/mL), DOX (10, 20, 50 μg/mL), or combinations of both. After 24 h, 0.5 mg/mL MTT solution was added and incubated for 2 more hours. The supernatant was removed, and 100 μL DMSO was added to dissolve the formazan crystals. Plates were gently shaken for 5 min, and absorbance was measured at 570 nm. Cell viability was expressed as a percentage relative to untreated controls.

Intracellular calcium levels in 4 T1 murine breast cancer cells were measured using the ratiometric fluorescent dye Fura‐2 AM (Thermo Fisher Scientific, F1225), following the method of Rodrigues and Ferraz (2021). Cells were seeded at 5 × 10^4^ cells/well on 96‐well plates or sterile coverslips and incubated overnight at 37°C in 5% CO_2_. A 1 mM stock solution of Fura‐2 AM was prepared in DMSO with 0.02% Pluronic F‐127. The working solution was diluted to 5 μM in HBSS containing 0.1% fatty acid‐free BSA. Cells were washed twice with HBSS, incubated with dye for 45 min in the dark, then washed three times and incubated for 30 min to complete de‐esterification. Fluorescence was measured using a microscope (340/380 nm excitation, 510 nm emission) or microplate reader. The 340/380 nm ratio was used to assess intracellular Ca^2+^ levels, normalized to baseline. Imaging was performed with a Leica DM‐IRB inverted microscope, and fluorescence intensity was analyzed using ImageJ/FIJI software. For quantitative analysis, at least five non‐overlapping fields per condition were selected to ensure representative sampling. Fluorescence images (Fluo‐4 channel) were converted to 8‐bit grayscale, and a consistent thresholding criterion was applied across all groups using the “Auto Threshold” (Otsu method) to minimize bias. Regions of interest (ROIs) were manually defined to exclude background and debris, and mean fluorescence intensity was calculated for each ROI. Data were normalized to the Sham control group and expressed as relative fluorescence (%).

### 2.6 Tumor Imaging Analysis of BALB/c Mice

To evaluate in vivo tumor growth and metastasis, bioluminescence and fluorescence imaging were performed using the IVIS Lumina II system (PerkinElmer, USA), following Luker and Luker (2008). The 4 T1 breast cancer cells used in this study were stably transduced with a luciferase reporter gene, enabling quantifiable bioluminescent signals in mice. At designated time points, mice received intraperitoneal injections of D‐luciferin (150 mg/kg; GoldBio, USA) 10–15 min before imaging to activate luciferase. Mice were anesthetized with 2%–3% isoflurane and placed on the imaging stage. Exposure time (1–5 min) and sensitivity were optimized for each scan. Imaging data were analyzed using Living Image software for signal quantification, background correction, and longitudinal tracking. Regions of interest (ROIs) were defined at the tumor site (subcutaneous flank) and distant organs (lungs, liver, lymph nodes) to assess metastasis. Total flux (photons/s/cm^2^/sr) was measured in each ROI to evaluate tumor burden. Signal intensities were statistically compared over time along with physiological parameters (body weight, blood pressure, heart rate) to determine the therapeutic effects of ZGE, DOX, and their combination.

### 2.7 IHC Analysis of 4 T1 Cells and Tumor Tissues in BALB/c Mice

Due to the limited availability of tumor tissue and the need to preserve spatial resolution, immunohistochemistry (IHC) was selected over biochemical assays. IHC enables semi‐quantitative analysis of protein expression within preserved tissue architecture, allowing precise localization of TRPM2, SOD2, TNF‐α, and cleaved caspase‐3. This approach is particularly advantageous in small animal models, where sample volume is constrained and multiplexed analysis is required (Prichard 2022). In this study, IHC analysis was performed on 4 T1 murine breast cancer cells and tumor tissues from BALB/c mice. 4 T1 cells were fixed with 4% paraformaldehyde, dehydrated, cleared, and embedded in paraffin. Sections (5 μm) were cut and mounted on Superfrost Plus slides. Antigen retrieval was conducted in 10 mM sodium citrate buffer (pH 6.0) at 95°C–100°C. Endogenous peroxidase activity was blocked with 3% hydrogen peroxide, and non‐specific binding was blocked with 1% BSA in PBS. Slides were incubated overnight at 4°C with rabbit polyclonal anti‐TRPM2 antibody (1:200), followed by HRP‐conjugated goat anti‐rabbit secondary antibody. DAB substrate was used for signal detection, and nuclei were counterstained with hematoxylin. Stained sections were mounted with DPX and examined under a light microscope (BX53, Olympus). TRPM2 expression was quantified using ImageJ software based on DAB‐positive area and optical density.

Tumor tissues were collected from anesthetized BALB/c mice after transcardial perfusion with 4% paraformaldehyde in PBS. Paraffin‐embedded sections (5 μm) were stained with hematoxylin and eosin (H&E) to assess morphology. For immunohistochemistry, sections were incubated with rabbit polyclonal antibodies (1:200) against TRPM2, SOD2, TNF‐α, and cleaved Caspase‐3. Detection was performed using the Novolink Polymer Detection System, including biotinylated secondary antibody and streptavidin‐HRP, followed by DAB visualization and hematoxylin counterstaining. Protein expression was quantified using ImageJ software. Negative controls included sections incubated with secondary antibody only to confirm specificity.

TRPM2, SOD2, TNF‐α, and cleaved Caspase‐3 expressions were visualized by IHC staining and imaged using a Leica DM‐IRB inverted microscope. Quantitative analysis of intensity was performed using ImageJ/FIJI software. For each treatment group (Sham, DOX, ZGE, ZGE + DOX), at least five randomly selected, non‐overlapping microscopic fields were analyzed to ensure statistical robustness and minimize sampling bias. Images were first converted to 8‐bit grayscale, and a consistent thresholding method was applied using the “Auto Threshold” function (Otsu algorithm) to delineate positively stained regions. Regions of interest (ROIs) were manually defined to exclude background and artifacts. The mean intensity of TRPM2, SOD2, TNF‐α, and cleaved Caspase‐3 staining within each ROI was measured and normalized to the Sham control group. Quantified data were expressed as relative expression (%), and statistical comparisons among groups were conducted.

### 2.8 Serum Biochemical Analysis of BALB/c Mice

To evaluate systemic responses and potential organ stress from treatment, serum biochemical markers were analyzed after 4 weeks of intervention. Whole blood was collected via cardiac puncture following anesthesia and euthanasia. Serum was isolated by centrifugation at 3000 × g for 10 min at 4°C and stored at −80°C. Quantitative analysis was performed using an automated biochemical analyzer (Hitachi 7600, Japan) following manufacturer protocols. The following markers were measured: BUN (renal function), CKMB (cardiac injury), LDH (cellular damage), GOT and GPT (hepatic function). Parameters were compared across experimental groups: Sham control (CON), DOX chemotherapy, ZGE alone, and ZGE + DOX combination.

### 2.9 Statistical Analysis

All data were expressed as mean ± standard error of the mean (SEM). Statistical analyses and graphing were performed using SigmaPlot 12.5 (Systat Software Inc., USA). Prior to group comparisons, biochemical markers (e.g., ALT, AST, BUN, CRE) and imaging data (e.g., IVIS fluorescence intensity) were tested for normality and homogeneity of variance. One‐way or two‐way ANOVA was used to assess differences among experimental groups. When a significant F value was obtained, the Student–Newman–Keuls post hoc test was applied to determine intergroup differences. Imaging quantification was performed using ImageJ (NIH, USA), with regions of interest (ROIs) selected and signal intensities extracted for analysis. Statistical significance was defined as *p* < 0.05.

## 3 Results

### 3.1 Antioxidant Capacity of ZGE

**FIGURE 3:**
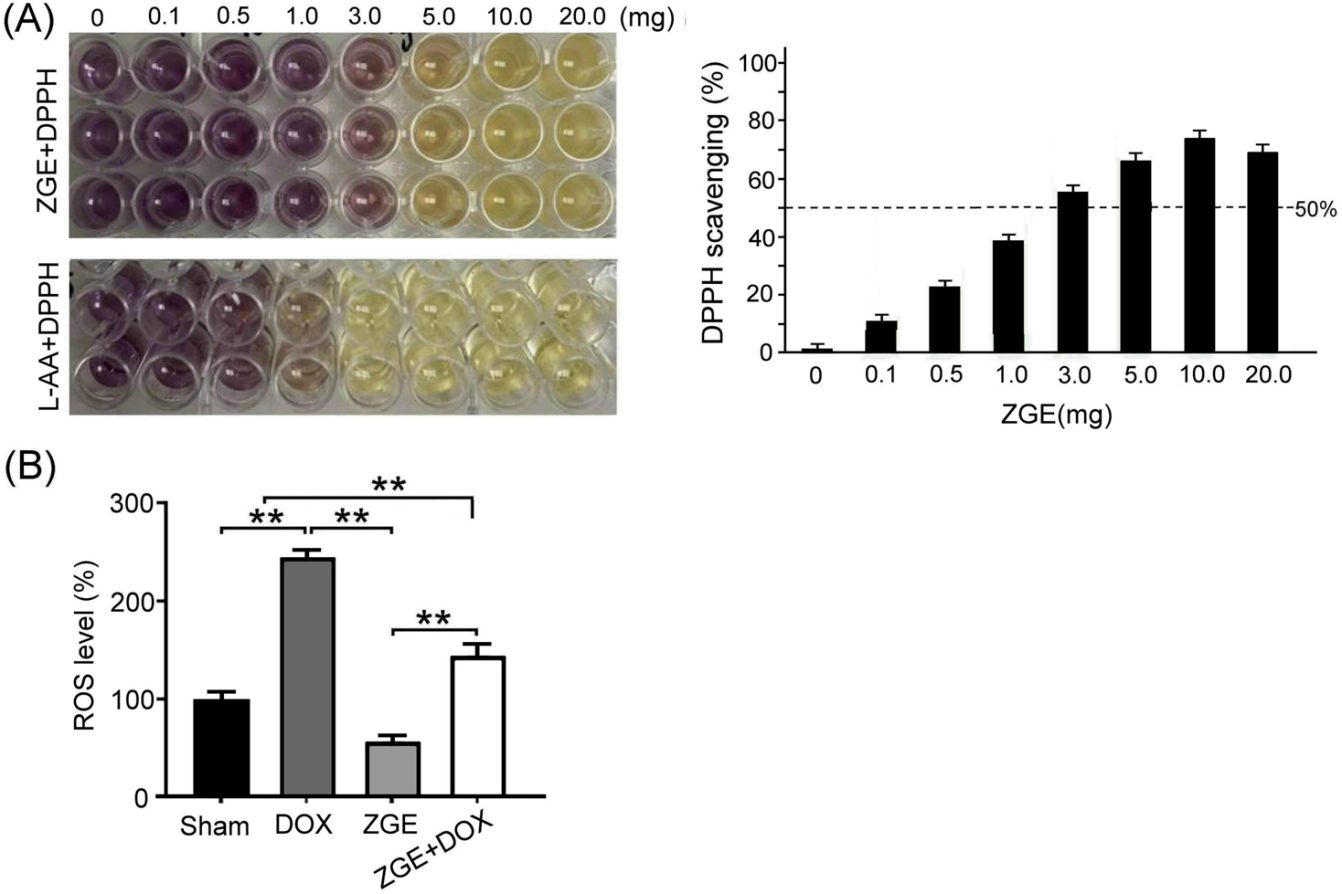
Antioxidant activity of ZGE evaluated by DPPH assay and ROS quantification. (A) ZGE shows dose‐dependent DPPH radical scavenging activity across concentrations (0–20.0 mg), with visible color fading in wells and quantified bar graph results. L‐ascorbic acid (L‐AA) serves as a positive control. (B) Intracellular ROS levels in 4 T1 cells under Sham, DOX, ZGE, and ZGE + DOX treatments. Data are mean ± SEM (*N* = 3); **p < 0.01 by one‐way ANOVA with Student–Newman–Keuls post‐test.

The antioxidant potential of ZGE was assessed using the DPPH radical scavenging assay. As shown in Figure 3A, upon mixing ZGE with DPPH solution, a visible color transition from deep purple to yellow was observed, indicating radical neutralization activity. This trend closely mirrored the positive control treated with L‐ascorbic acid (L‐AA). Quantitative absorbance measurements revealed a dose‐dependent increase in scavenging efficiency across the concentration range of 0.1 to 20.0 mg, with higher concentrations approaching 50% activity, highlighting the extract's substantial antioxidant capacity. These findings underscore the potential of ZGE as a natural antioxidant agent and provide a foundation for further pharmacological evaluation in cellular and in vivo models. Moreover, analysis of ZGE‐treated samples revealed a gradual increase in radical scavenging capacity with increasing treatment dose. The quantitative DPPH radical scavenging activity of ZGE corresponded well with the standard reference L‐AA. The reduction capacity measured for both ZGE and L‐AA demonstrated consistent trends, suggesting superior free radical scavenging performance of ZGE against oxidative damage.

As shown in Figure 3B, intracellular reactive oxygen species (ROS) levels were shown in 4 T1 cells under different treatment conditions. The DOX group exhibited markedly elevated ROS levels, while ZGE treatment alone significantly suppressed ROS accumulation. Notably, co‐treatment with ZGE and DOX attenuated DOX‐induced oxidative stress, suggesting a protective antioxidant role for ZGE. Statistical analysis confirms significant differences (*p* < 0.01) between groups.

### 3.2 Cytotoxicity of ZGE and DOX

**FIGURE 4:**
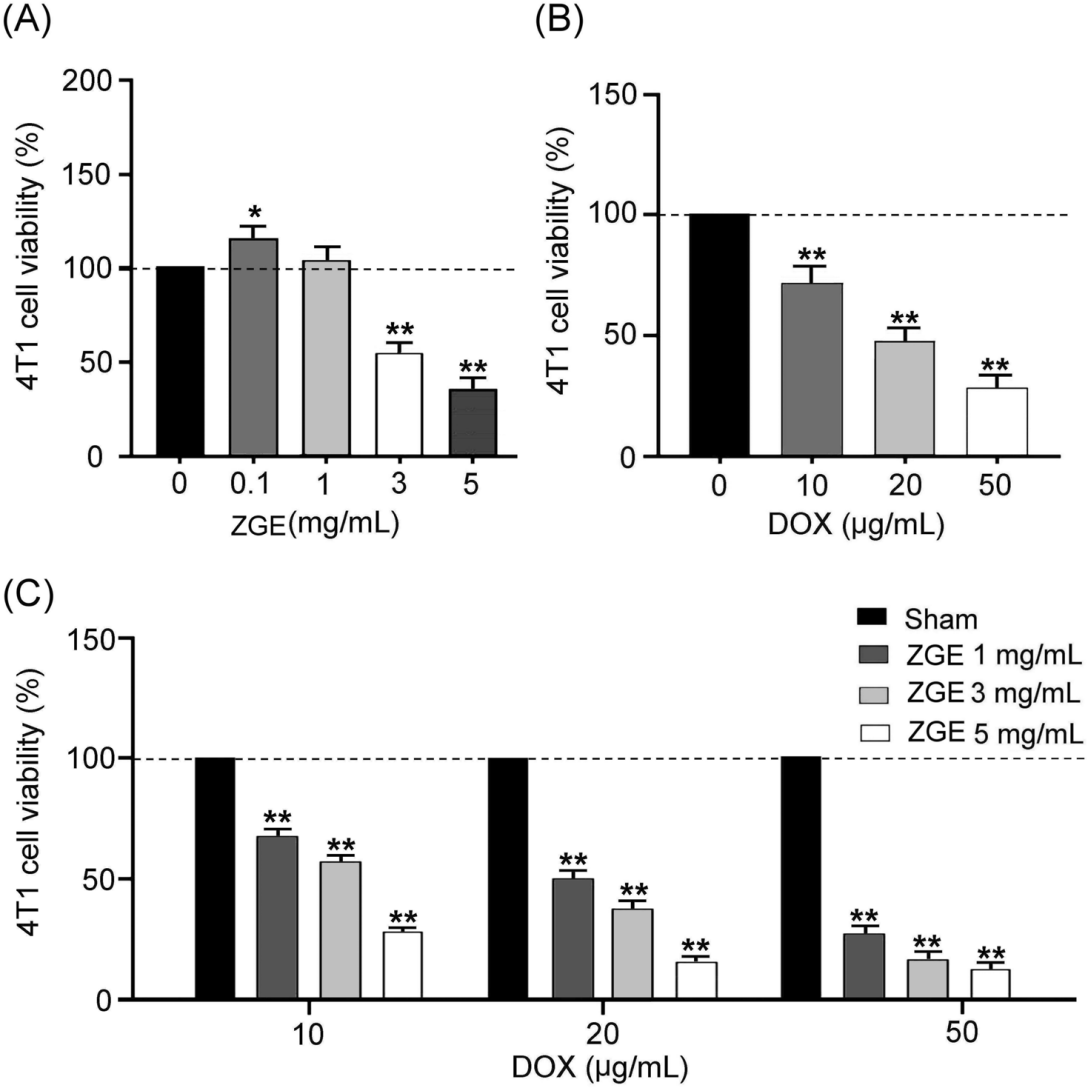
Cytotoxic effects of ZGE and DOX on 4 T1 cells. (A) ZGE (0–5 mg/mL) reduced cell viability in a dose‐dependent manner after 24 h. (B) DOX (10–50 μg/mL) significantly decreased viability. (C) Co‐treatment with ZGE and DOX further enhanced cytotoxicity compared to DOX alone. Data are mean ± SD (*N* = 3); *p < 0.05, **p < 0.01 by two‐way ANOVA with Student–Newman–Keuls post‐test.

To evaluate the cytotoxic potential of ZGE, 4 T1 murine breast cancer cells were treated with increasing concentrations of ZGE (0, 0.1, 1, 3, and 5 mg/mL) for 24 h. As shown in Figure 4A, ZGE induced a dose‐dependent reduction in 4 T1 cell viability, with significant cytotoxic effects observed at ≥ 3 mg/mL (*p* < 0.01–0.05). Interestingly, a slight increase in viability was noted at 0.1 mg/mL (*p* < 0.05), suggesting a biphasic response. Separately, DOX treatment at 10, 20, and 50 μg/mL significantly suppressed 4 T1 cell viability in a concentration‐dependent manner (Figure 4B,p < 0.01 for all doses). Combined treatment with ZGE (1, 3, and 5 mg/mL) and DOX (10, 20, and 50 μg/mL) further enhanced cytotoxicity (Figure 4C), with viability decreasing across all combinations. The most pronounced suppression was observed at 50 μg/mL DOX + 5 mg/mL ZGE (*p* < 0.01), indicating a synergistic or additive interaction. These results suggest that ZGE not only exerts independent anticancer effects but also potentiates the efficacy of DOX in combination therapy.

### 3.3 ZGE Mitigates DOX‐Induced TRPM2 Upregulation in 4 T1 Cells

**FIGURE 5:**
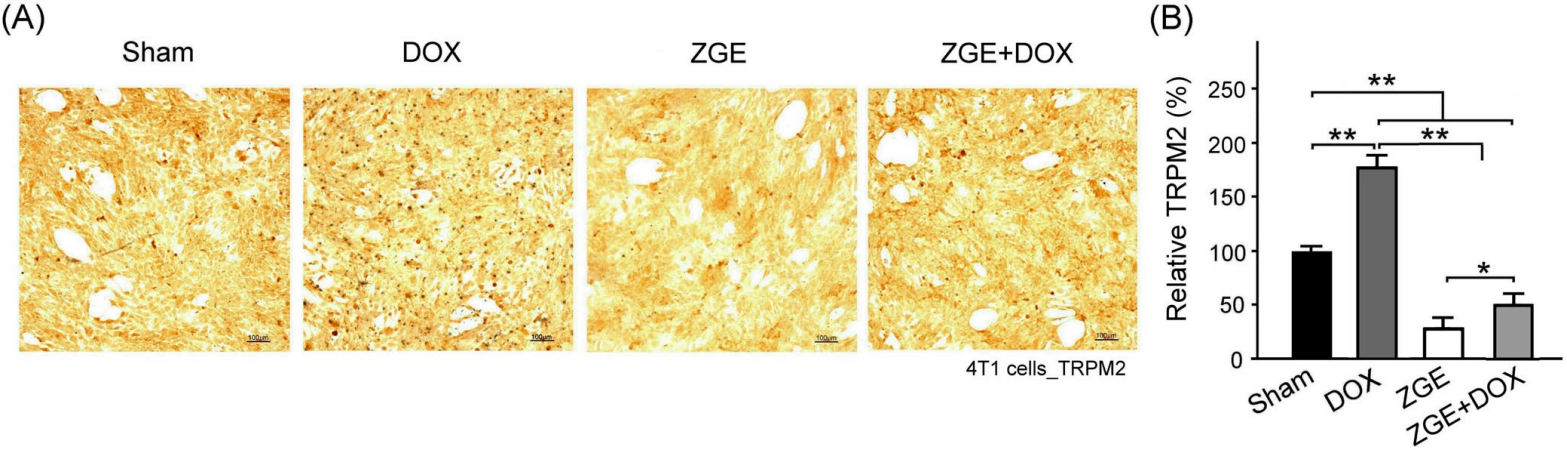
ZGE suppresses DOX‐induced TRPM2 expression in 4 T1 cells. (A) IHC staining shows TRPM2 levels under Sham, DOX, ZGE, and ZGE + DOX treatments. DOX increased TRPM2 expression; ZGE and ZGE + DOX reduced it, with ZGE + DOX showing slightly higher levels than ZGE alone. Scale bar = 100 μm. (B) Bar graph quantifies relative TRPM2 expression (%). DOX significantly elevated TRPM2 vs. Sham (**p < 0.01); ZGE and ZGE + DOX both reduced TRPM2 vs. DOX (**p < 0.01), with ZGE + DOX slightly higher than ZGE (*p < 0.05). Data are mean ± SEM (*N* = 3); one‐way ANOVA with Student–Newman–Keuls post‐test.

To investigate the regulatory effect of ZGE on TRPM2 expression under chemotherapeutic stress, 4 T1 cells were treated with DOX, ZGE, or their combination. IHC analysis revealed that DOX markedly increased TRPM2 signal intensity compared to the Sham group, indicating a stress‐induced upregulation of TRPM2 (Figure 5A). Treatment with ZGE alone reduced TRPM2 expression, while co‐treatment with ZGE and DOX significantly suppressed DOX‐induced TRPM2 elevation. Notably, TRPM2 levels in the ZGE + DOX group remained slightly higher than those in the ZGE group.

Quantitative analysis confirmed these observations (Figure 5B). DOX treatment significantly elevated TRPM2 levels (*p* < 0.01 vs. Sham), whereas ZGE and ZGE + DOX both reduced TRPM2 expression (*p* < 0.01 vs. DOX, respectively). The ZGE + DOX group showed a modest but statistically significant increase in TRPM2 compared to ZGE alone (*p* < 0.05), suggesting that while ZGE effectively counteracts DOX‐induced TRPM2 upregulation, the combined treatment does not fully restore TRPM2 suppression to the level achieved by ZGE alone.

### 3.4 ZGE Alleviates DOX‐Induced Intracellular Calcium Overload in 4 T1 Cells

**FIGURE 6:**
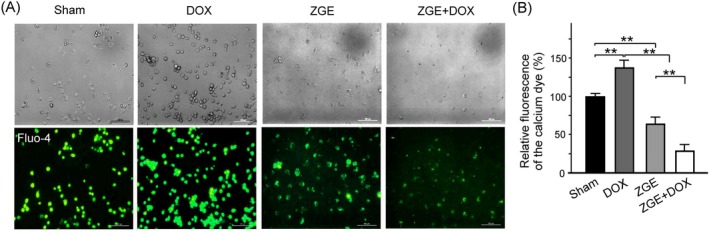
ZGE reduces DOX‐induced intracellular calcium accumulation in 4 T1 cells. (A) Fluo‐4 fluorescence images show calcium levels under Sham, DOX, ZGE, and ZGE + DOX treatments. DOX increased green fluorescence; ZGE and ZGE + DOX reduced calcium signals. Scale bar = 100 μm. (B) Bar graph quantifies relative Fluo‐4 fluorescence (%). DOX significantly elevated calcium uptake vs. Sham (**p < 0.01); ZGE and ZGE + DOX both reduced fluorescence vs. DOX (**p < 0.01), with statistical comparisons shown. Data are mean ± SEM (*N* = 3); **p < 0.01 by one‐way ANOVA with Student–Newman–Keuls post‐test.

To assess the effect of ZGE on intracellular calcium dynamics under DOX‐induced stress, 4 T1 cells were labeled with the calcium‐sensitive dye Fluo‐4 and examined by fluorescence microscopy. As shown in Figure 6A, DOX treatment markedly increased green fluorescence intensity, indicating elevated intracellular calcium levels. In contrast, ZGE‐treated cells exhibited reduced fluorescence, and co‐treatment with ZGE and DOX further attenuated the DOX‐induced calcium accumulation. Quantitative analysis of Fluo‐4 fluorescence confirmed these observations (Figure 6B). DOX significantly increased calcium dye uptake compared to the Sham group (*p* < 0.01), while both ZGE and ZGE + DOX treatments significantly reduced fluorescence intensity (*p* < 0.01 vs. DOX). These results suggest that ZGE effectively mitigates DOX‐induced calcium overload in 4 T1 cells, potentially contributing to its protective effects under chemotherapeutic stress.

### 3.5 ZGE Enhances the Antitumor Efficacy of Doxorubicin In Vivo

**FIGURE 7:**
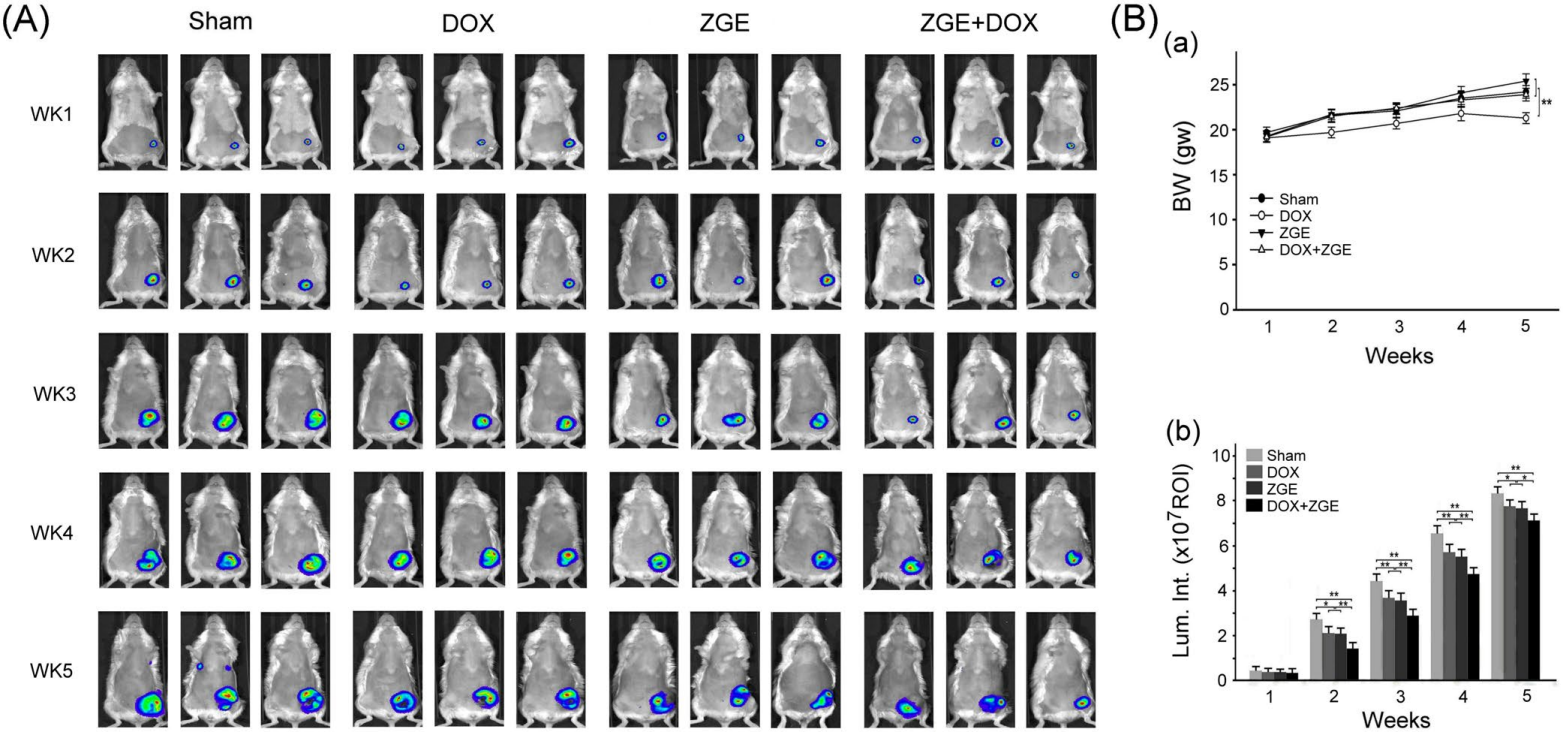
ZGE enhances the antitumor effect of DOX and alleviates body weight loss in 4 T1 tumor‐bearing mice. (A) Weekly bioluminescent images (Week 1–5) show tumor signals in Sham, DOX, ZGE, and ZGE + DOX groups. DOX increased abdominal luminescence; ZGE + DOX further reduced signal intensity. (B) Quantitative analysis of (a) body weight and (b) luminescence intensity. DOX decreased body weight and increased tumor signal; ZGE co‐treatment mitigated weight loss and enhanced tumor suppression. Data are mean ± SEM (*N* = 8); *p < 0.05, **p < 0.01 by two‐way ANOVA with Student–Newman–Keuls post‐test.

To assess the longitudinal effects of ZGE and DOX on tumor progression and host physiology, bioluminescent imaging and body weight monitoring were conducted weekly in 4 T1 tumor‐bearing mice. As shown in Figure 7A, Sham mice exhibited a gradual increase in abdominal luminescence from Week 1 to Week 5, indicating progressive tumor growth. DOX‐treated mice displayed markedly reduced luminescence signals throughout the study period, suggesting effective tumor suppression. Notably, combined treatment with ZGE and DOX further decreased luminescence intensity compared to DOX alone (*p* < 0.01), indicating a synergistic antitumor effect. Quantitative analysis of luminescence intensity (Figure 7Bb) revealed that the ZGE + DOX group exhibited the lowest bioluminescent signal among all groups, while ZGE alone produced a moderate reduction relative to Sham. These findings suggest that ZGE potentiates the tumor‐inhibitory action of DOX. In parallel, body weight measurements (Figure 7Ba) showed that DOX treatment led to significant weight loss over time (*p* < 0.01 vs. Sham), reflecting systemic toxicity. Importantly, co‐treatment with ZGE significantly attenuated DOX‐induced weight loss (*p* < 0.01 vs. DOX), indicating a protective effect on host physiology. Together, these results demonstrate that ZGE not only enhances the antitumor efficacy of DOX but also mitigates its adverse effects, supporting its potential as a complementary therapeutic agent in breast cancer treatment.

### 3.6 ZGE Modulates Oxidative Stress, Inflammation, and Apoptosis Markers in DOX‐Treated Tumor Tissues

**FIGURE 8:**
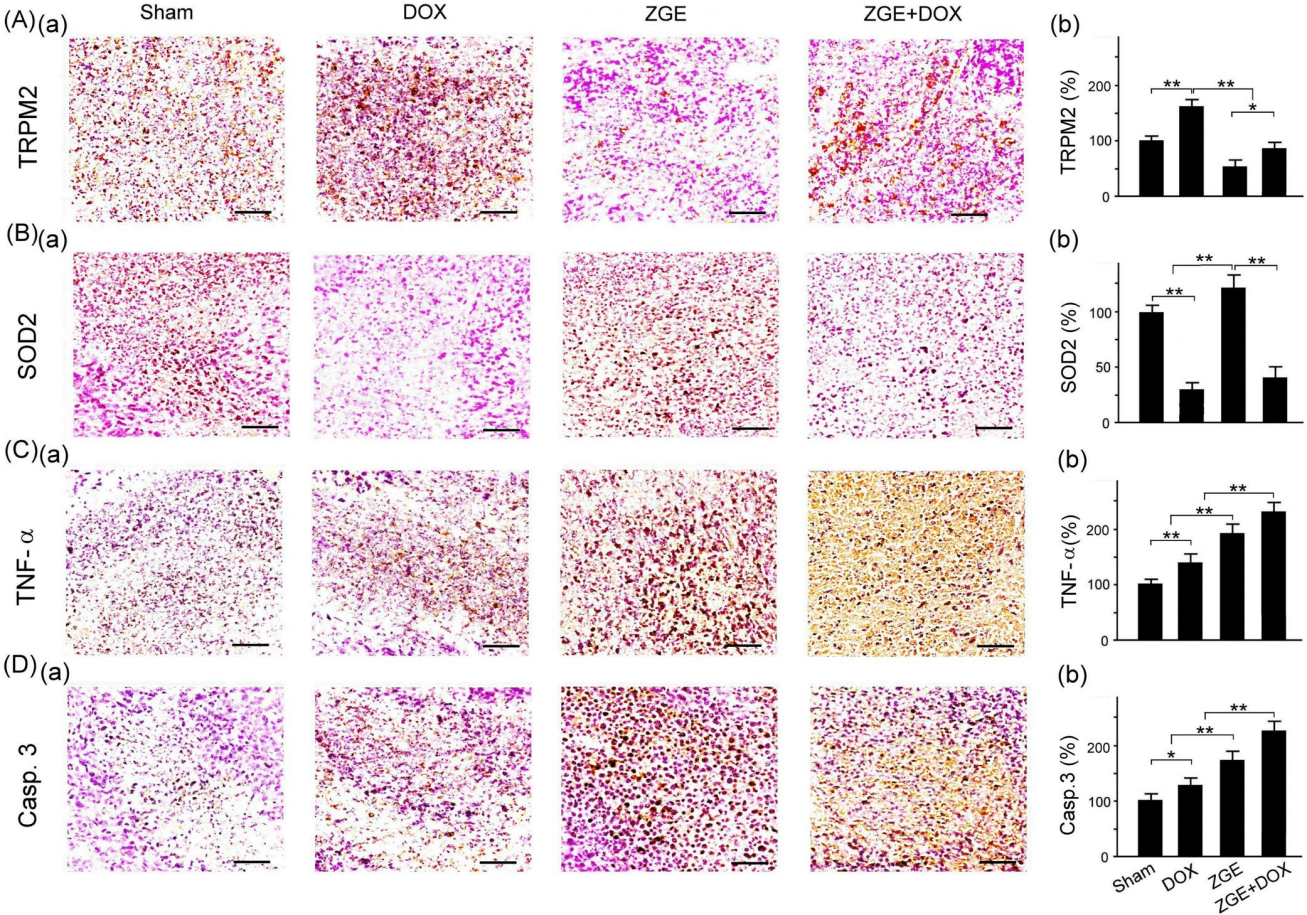
Effects of ZGE and DOX on TRPM2, SOD2, TNF‐α, and cleaved caspase‐3 expression in tumor tissues. (A–D) Immunohistochemical staining (a) and quantification (b) of TRPM2 (A), SOD2 (B), TNF‐α (C), and cleaved caspase‐3 (D) in Sham, DOX, ZGE, and ZGE + DOX groups. Data are mean ± SEM (*N* = 8); *p < 0.05, **p < 0.01 by one‐way ANOVA with Student–Newman–Keuls post‐test. Scale bar = 50 μm.

IHC staining and quantitative analysis revealed distinct regulatory effects of ZGE and DOX on key molecular markers associated with oxidative stress, inflammation, and apoptosis. TRPM2, a redox‐sensitive, non‐selective cation channel implicated in ROS‐mediated calcium influx and cell death, exhibited significantly elevated expression in the DOX group, indicating enhanced oxidative stress and TRPM2 activation. Expression levels were progressively lower in Sham, ZGE + DOX, and ZGE groups (Figure 8A), suggesting that ZGE may attenuate DOX‐induced TRPM2 upregulation, potentially through antioxidant mechanisms.

SOD2, a mitochondrial superoxide dismutase critical for detoxifying superoxide radicals, showed the highest expression in the ZGE group, followed by Sham and ZGE + DOX, with the lowest levels observed in DOX‐treated tissues (Figure 8B). This pattern supports the hypothesis that ZGE enhances endogenous antioxidant defenses, possibly counteracting DOX‐induced mitochondrial oxidative damage. The partial restoration of SOD2 expression in the ZGE + DOX group further suggests a protective role of ZGE when co‐administered with DOX.

TNF‐α, a pro‐inflammatory cytokine involved in tumor progression and immune modulation, was markedly elevated in the ZGE + DOX group, followed by the ZGE and DOX groups, with minimal expression in Sham tissues (Figure 8C). The synergistic increase in TNF‐α in the ZGE + DOX group may reflect enhanced immune activation or stress signaling, potentially contributing to tumor suppression via inflammatory‐mediated apoptosis or immune recruitment.

Cleaved caspase‐3, a terminal effector of the apoptotic cascade, showed the highest expression in the ZGE + DOX group, followed by the ZGE and DOX groups, with the lowest levels in Sham (Figure 8D).

These results indicate that ZGE not only potentiates DOX‐induced apoptosis but may also independently promote caspase‐3 activation. The concurrent elevation of TNF‐α and caspase‐3 in the ZGE + DOX group suggests a coordinated enhancement of inflammatory and apoptotic pathways, possibly contributing to improved antitumor efficacy. Collectively, these findings demonstrate that ZGE modulates multiple stress‐responsive pathways in tumor tissues, attenuating oxidative damage (via TRPM2 and SOD2), amplifying inflammatory signaling (via TNF‐α), and promoting apoptosis (via caspase‐3), particularly when combined with DOX treatment.

### 3.7 ZGE Attenuates DOX‐Induced Renal, Cardiac, and Hepatic Toxicity in Blank and Tumor‐Bearing Mice

**FIGURE 9:**
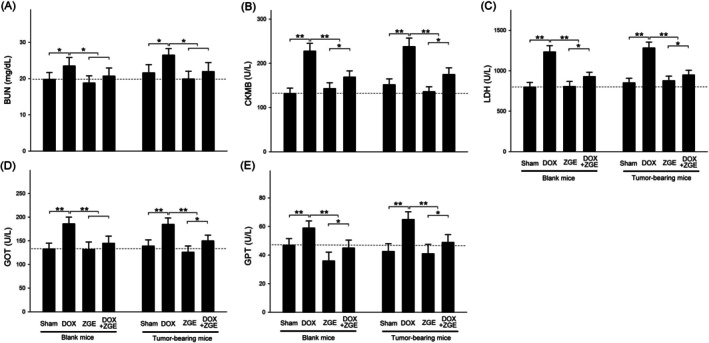
Effects of DOX and ZGE on serum biochemical markers in blank and tumor‐bearing mice. (A–E) Serum levels of BUN (A), CKMB (B), LDH (C), GOT (D), and GPT (E) were assessed in mice under Sham, DOX, ZGE, and DOX + ZGE treatments. In both blank and tumor‐bearing mice, DOX elevated all five markers; ZGE co‐treatment reduced these elevations. Data are mean ± SEM (*N* = 8); *p < 0.05, **p < 0.01 by two‐way ANOVA with Student–Newman–Keuls post‐test.

To assess the systemic toxicity induced by DOX and the potential protective role of ZGE, serum biochemical markers were evaluated in both blank and tumor‐bearing mice. In blank mice, DOX administration significantly increased blood urea nitrogen (BUN), creatine kinase‐MB (CKMB), lactate dehydrogenase (LDH), glutamate oxaloacetate transaminase (GOT), and glutamate pyruvate transaminase (GPT) levels compared to the Sham group (Figure 9A–E). Elevated BUN reflects impaired renal filtration function, while increased CKMB and LDH are indicative of myocardial damage and cellular injury. The rise in GOT and GPT levels further suggests hepatocellular stress and compromised liver function.

ZGE treatment alone did not significantly alter these parameters relative to Sham, indicating its safety and lack of intrinsic toxicity. Importantly, co‐treatment with ZGE (DOX + ZGE group) markedly attenuated the DOX‐induced elevations in all five markers, suggesting that ZGE exerts a protective effect against DOX‐mediated renal, cardiac, and hepatic injury. The reduction in CKMB and LDH levels implies that ZGE may stabilize cardiomyocyte membranes and reduce oxidative damage, while the normalization of GOT and GPT levels points to hepatoprotective activity, possibly through modulation of inflammatory and antioxidant pathways.

In tumor‐bearing mice, DOX‐induced toxicity was further exacerbated, with significantly higher levels of BUN, CKMB, LDH, GOT, and GPT compared to sham controls. This reflects the compounded physiological burden of both tumor progression and chemotherapeutic stress. ZGE alone maintained biochemical parameters within normal ranges, reinforcing its non‐toxic profile in tumor‐bearing hosts. Notably, the DOX + ZGE group exhibited substantial reductions in all five markers compared to the DOX group, demonstrating that ZGE retains its protective efficacy even under tumor‐bearing conditions.

These results collectively indicate that ZGE mitigates DOX‐induced multi‐organ toxicity through mechanisms that may involve antioxidant defense, membrane stabilization, and suppression of inflammatory mediators. The consistent protective effects observed in both blank and tumor‐bearing models underscore ZGE's potential as a safe and effective adjuvant in DOX‐based chemotherapy regimens.

## 4 Discussion

The present study provides compelling evidence that ZGE, rich in bioactive compounds such as zerumbone, exerts both antitumor and organ‐protective effects in a murine breast cancer model. Through a combination of in vitro and in vivo analyses, we demonstrate that ZGE enhances the efficacy of DOX while mitigating its systemic toxicity, suggesting its potential as a natural adjuvant in cancer therapy.

Ginger has emerged as a promising adjunctive therapy for alleviating chemotherapy‐related side effects in breast cancer patients (Kim et al. 2022). Daily administration of ginger extract significantly reduced nausea and vomiting in patients undergoing chemotherapy (Arslan and Ozdemir 2015; Bossi et al. 2017; Marx et al. 2017; Pillai et al. 2011). Aromatherapy using ginger essential oils further improved overall well‐being and appetite during treatment (Lua et al. 2015). Ginger also exhibits anti‐breast cancer properties (Li et al. 2017). Moreover, our GC–MS analysis revealed that ZGE is rich in zerumbone (Figure 1), a naturally occurring monocyclic sesquiterpene known for its anti‐inflammatory, anticancer, hepatoprotective, and antidiabetic pharmacological activities (Dehghan et al. 2021; Radaei et al. 2020; Wang et al. 2016). Zerumbone also exhibits pro‐apoptotic, anti‐inflammatory, and redox‐modulating properties (Jalili‐Nik et al. 2020). In our study, ZGE exhibited robust antioxidant capacity, as evidenced by dose‐dependent DPPH radical scavenging (Figure 3A) and suppression of intracellular ROS accumulation in 4 T1 cells (Figure 3B). These findings are consistent with previous reports that zerumbone can either promote ROS‐mediated apoptosis in malignant cells or protect normal tissues from oxidative damage via thiol‐dependent mechanisms (Deorukhkar et al. 2015). Importantly, ZGE demonstrated significant cytotoxicity against 4 T1 cells and potentiated DOX‐induced cell death (Figure 4). The combination treatment resulted in a marked reduction in cell viability (Figure 4C) and tumor bioluminescence (Figure 7), indicating synergistic antitumor activity. This effect was accompanied by enhanced expression of cleaved caspase‐3 and TNF‐α, suggesting activation of apoptotic and inflammatory pathways (Figure 8). Notably, ZGE also upregulated SOD2, an endogenous antioxidant enzyme, further supporting its role in redox homeostasis.

A key mechanistic insight from this study is the modulation of TRPM2, a redox‐sensitive calcium‐permeable channel implicated in cancer cell survival, mitochondrial function, and chemoresistance (Ali et al. 2023; Miller 2019). TRPM2 activation has been shown to support tumor growth by maintaining mitochondrial integrity and suppressing ROS accumulation. In our model, DOX treatment significantly upregulated TRPM2 expression (Figure 5) and induced intracellular calcium overload (Figure 6), consistent with previous findings in laryngeal squamous carcinoma and neuroblastoma cells (Chen et al. 2025; Hirschler‐Laszkiewicz et al. 2018). Pharmacological inhibition of TRPM2 significantly suppresses cancer cell proliferation and increases DNA damage (McHugh et al. 2003). TRPM2 inhibition has also been shown to induce DNA damage and apoptosis in both TNBC and estrogen receptor‐positive breast cancer models (Koh et al. 2015). ZGE effectively counteracted these effects, downregulating TRPM2 and restoring calcium balance, thereby sensitizing tumor cells to DOX‐induced oxidative stress and apoptosis. The dual role of TRPM2 in cancer progression and organ injury underscores its relevance as a therapeutic target. While TRPM2 activation may confer survival advantages to tumor cells, its overactivation in non‐cancerous tissues contributes to cytotoxicity and inflammation. ZGE's ability to modulate TRPM2 expression in both contexts suggests a context‐dependent regulatory effect, enhancing cytotoxicity in malignant cells while preserving normal tissue integrity. Furthermore, the IHC data revealed that ZGE treatment led to coordinated regulation of multiple molecular markers. The downregulation of TRPM2 and calcium overload, coupled with increased expression of cleaved caspase‐3 and TNF‐α, points to a multifactorial mechanism involving oxidative stress modulation, calcium signaling, and apoptotic induction. These effects were more pronounced in the ZGE + DOX group, indicating a synergistic interaction that amplifies therapeutic efficacy while minimizing adverse effects. Taken together, our findings suggest that ZGE exerts its therapeutic effects through a complex interplay of antioxidant activity, TRPM2‐mediated calcium signaling, and apoptotic regulation. Its ability to enhance DOX efficacy while protecting vital organs positions ZGE as a promising candidate for integrative cancer therapy. The phytochemical richness of ZGE, particularly its zerumbone content, may underlie its multi‐targeted actions, offering advantages over single‐compound interventions.

Beyond its antitumor effects, ZGE exhibited notable organ‐protective properties. DOX is known to induce multi‐organ toxicity, including nephrotoxicity, hepatotoxicity, and cardiotoxicity, primarily through oxidative stress and mitochondrial dysfunction (Yu et al. 2023). In our study, ZGE significantly reduced serum levels of BUN, CKMB, LDH, GOT, and GPT, indicating protection of renal, cardiac, and hepatic function (Figure 9). These findings are supported by prior studies showing that TRPM2 inhibition can alleviate cisplatin‐induced acute kidney injury and ischemia–reperfusion damage via autophagy modulation and ROS suppression (Chen et al. 2025). To improve therapeutic outcomes and minimize side effects, combinatorial treatments involving doxorubicin and other agents are increasingly studied. Quercetin combined with doxorubicin, for instance, enhances chemotherapeutic efficacy and reduces toxicity, suggesting that natural compounds may help overcome multidrug resistance (Mattioli et al. 2023; Rahman et al. 2023). Our previous studies demonstrated that traditional Chinese herbal medicines such as B307 and Guilu Erxian Jiao could alleviate doxorubicin‐induced cardiotoxicity in mice by suppressing oxidative stress, inflammation, and apoptosis (Lien et al. 2015, 2021). Other natural agents—including cantharides complex, *Anoectochilus roxburghii*, and *Dendrobium fimbriatum* Hook extracts—enhanced doxorubicin's efficacy against breast cancer while mitigating cardiotoxicity and retinopathy (Cheng et al. 2023, 2024; Lu et al. 2021). Probiotic supplementation also effectively reduced doxorubicin‐induced oxidative stress and inflammation (Cheng et al. 2025). Future studies should focus on elucidating the pharmacokinetics and bioavailability of ZGE constituents, as well as their interactions with conventional chemotherapeutics.

## 5 Conclusions

In conclusion, ZGE emerges as a promising plant‐derived adjuvant with dual antitumor and cytoprotective properties. By modulating oxidative stress and TRPM2 signaling pathways, ZGE enhances the therapeutic efficacy of DOX while significantly reducing its systemic toxicity. In a murine breast cancer model, ZGE demonstrated potent antioxidant and cytotoxic activities, suppressed TRPM2‐mediated oxidative stress, and promoted apoptosis via caspase‐3 activation. Notably, co‐administration of ZGE attenuated DOX‐induced renal, cardiac, and hepatic damage, as evidenced by normalized serum biochemical markers. These findings suggest that ZGE not only potentiates the antitumor effects of DOX but also safeguards non‐target organs, thereby improving the overall therapeutic index. Given its favorable safety profile and multi‐targeted mechanisms of action, ZGE holds strong potential as a natural adjuvant in DOX‐based chemotherapy. Further studies are warranted to elucidate its pharmacokinetics, bioavailability, and clinical applicability in integrative cancer treatment strategies.

## Author Contributions

**Li‐Yu Su:** data curation (equal), methodology (equal), writing original draft (equal). **Liang‐Chuan Lai:** conceptualization (equal), supervision (equal), review and editing (equal). **Chi‐Feng Cheng:** formal analysis (equal), investigation (equal), visualization (equal). **Chia‐Ying Lien:** data curation (equal), methodology (equal). **Ming‐Chung Lee:** software (equal), visualization (equal). **Wu‐Chang Chuang:** data curation (equal), visualization (equal). **Chung‐Hsin Wu:** supervision (equal), project administration (equal), resources (equal), review and editing (equal).

## Conflicts of Interest

The authors declare no conflicts of interest.

## Data Availability

The data that support the findings of this study are available on request from the corresponding author. The data are not publicly available due to privacy or ethical restrictions.
